# Focussing both eyes on health outcomes: revisiting cataract surgery

**DOI:** 10.1186/1471-2318-12-50

**Published:** 2012-09-03

**Authors:** Jennifer C Davis, Heather McNeill, Michael Wasdell, Susan Chunick, Stirling Bryan

**Affiliations:** 1 Centre for Clinical Epidemiology & Evaluation, Vancouver Coastal Health Research Institute, Vancouver, Canada; 2 School of Population and Public Health, Centre for Clinical Epidemiology and Evaluation VCH Research Institute, The University of British Columbia, Vancouver, Canada; 3 Department of Evaluation & Research Services, Fraser Health Authority, Surrey, Canada

**Keywords:** Cataract surgery, Visual function, Health outcomes

## Abstract

**Background:**

The appropriateness of cataract surgery procedures has been questioned, the suggestion being that the surgery is sometimes undertaken too early in the disease progression. Our three study questions were: What is the level of visual impairment in patients scheduled for cataract surgery? What is the improvement following surgery? Given the thresholds for a minimal detectable change (MDC) and a minimal clinically important difference (MCID), do gains in visual function reach the MDC and MCID thresholds?

**Methods:**

The sample included a prospective cohort of cataract surgery patients from four Fraser Health Authority ophthalmologists. Visual function (VF-14) was assessed pre-operatively and at seven weeks post-operatively. Two groups from this cohort were included in this analysis: ‘all first eyes’ (cataract extraction on first eye) and ‘both eyes’ (cataract removed from both eyes). Descriptive statistics, change scores for VF-14 for each eye group and proportion of patients who reach the MDC and MCID are reported.

**Results:**

One hundred and forty-two patients are included in the ‘all first eyes’ analyses and 55 in the ‘both eyes’ analyses. The mean pre-operative VF-14 score for the ‘all first eyes’ group was 86.7 (on a 0–100 scale where 100 is full visual function). The mean change in VF-14 for the 'both eyes' group was 7.5. Twenty-three percent of patients achieved improvements in visual function beyond the MCID threshold and 35% saw improvement beyond the MDC.

**Conclusions:**

Neither threshold level for MDC or MCID for the VF-14 scale was achieved for a majority of patients. A plausible explanation for this is the very high levels of pre-operative visual functioning.

## Background

The development of age-related cataracts is an inevitable part of ageing for many people and without effective treatment it is one of the leading causes of blindness worldwide [1]. The widely acknowledged standard of care is cataract extraction, typically performed first in the lowest functioning eye. Compelling research evidence supports the efficacy and cost-effectiveness of cataract extraction when performed in patients with poor baseline visual acuity [2-4]. Hence, it is not surprising that cataract removal, a 19-minute ambulatory surgery [5], is one of the most frequently performed surgical procedures in the developed world [6].

However, the appropriateness of some cataract surgery procedures has been questioned repeatedly, the suggestion being that the surgery is sometimes undertaken at too early a stage in the disease progression [7-9]. This concern is based on reports of wide variations in post-surgery outcomes and estimates of health gain [10]. Low thresholds for cataract surgery in a Canadian setting were demonstrated by Wright et al. in their Regional Evaluation of Surgical Indications and Outcomes (RESIO) study [11]. Thirty-two percent of RESIO patients scheduled for first cataract surgery had a pre-operative visual function score of 90 or higher on the visual functioning VF-14 scale (where 100 is full visual functioning). With such high levels of visual function, the scope for improvements in functioning is very limited. Therefore, some 10 years after publication of the RESIO data, we revisit cataract surgery thresholds and outcomes in Canada. Black and colleagues conducted a study in the UK that ascertained whether there was overutilization of cataract surgery in England [12]. Although the notable reduction in the visual function threshold for cataract surgery was due to improvements in the provision of cataract surgery, methodological challenges in measuring post surgical outcomes rendered it impossible to conclude whether overutilization was indeed occurring [12]. To our knowledge, there is no such recent work conducted in Canada. As such, this study seeks to explore visual impairment thresholds for cataract surgery and levels of improvement seen following cataract surgery in Canada. Further, we explore factors relating to variation in thresholds and outcomes relating to cataract surgery such as the unit of analysis.

This paper reports data collected for an evaluation of cataract surgery outcomes conducted at Fraser Health Authority ophthalmology practices in British Columbia between April 2009 and March 2010. Supplementary funding to Fraser Health Authority for an expansion of cataract surgery services is the context for the analysis reported in this paper. As the data were collected for the service evaluation of cataract surgery outcomes, this study did not require ethical review by the Fraser Health Authority Research Ethics Board.

The primary study questions are:

1 What level of visual impairment was seen in patients scheduled for cataract surgery?

2 What level of improvement in visual function was seen following cataract surgery?

On the latter issue, most previous work, including the RESIO study, assessed either the visual functioning gains after first eye surgery only [11] or after second eye surgery only [13,14]. The work reported here takes the view that cataract surgery should be defined as treatment of the person (i.e. surgery on both eyes) and not a single eye, given that sight in both eyes is important for health-related quality of life.

Recently, VF-14 thresholds for a minimal detectable change (MDC) and a minimal clinically important difference (MCID) were established [15]. MDC is the threshold for distinguishing between actual change and measurement error, estimated to be 10.81 on the VF-14 scale [15,16]. MCID is the threshold that indicates the minimum change in score necessary for a patient to experience a clinically important improvement, estimated as 15.57 for VF-14 [15,16]. Given these new thresholds, our third study question is:

3 Did gains in visual function following cataract surgery reach the MDC and MCID thresholds, and what are the implications of these thresholds for clinical practice in Canada?

## Methods

### Study design and sample

**Figure 1:**
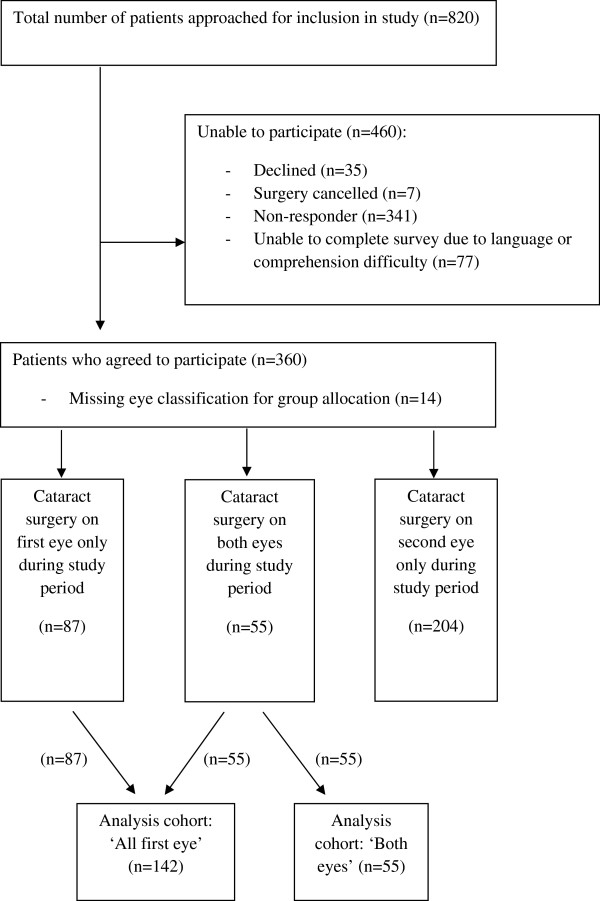
Flow of study subjects.

Data were collected on a population-based prospective cohort of ophthalmology patients listed for cataract surgery in the Fraser Health Authority in British Columbia between April 1, 2009 and March 31, 2010. The study design was a non-experimental pre-post-test design. Data were collected before surgery and at seven weeks post-cataract surgery to allow pre-operative visual function and visual acuity to be described and variation in patient outcomes to be explored. Figure 1 illustrates the distribution of the patient sample from which the data for this paper have been derived. Only 35 patients (8%) actively refused to take part in the evaluation. Within the study period, 87 patients received surgery on their first eye only and 55 had cataracts removed from both eyes.

### Measurements

At baseline, data were collected on:

• Visual function (VF-14) [17]

• Best corrected visual acuity (Snellen Fractions) [18]

• Clinical variables: glare, ocular comorbidities, eye surgery (first, second or both eyes)

• Indications for surgery (i.e., ‘significant cataract’, ‘needs better vision’ and ‘other medical indication’)

• Age and sex

Based on clinical considerations regarding the degree of severity of visual impairment, three Snellen visual acuity categories were used to reflect the visual acuity reported by the ophthalmologist: ≤0.1 (lowest acuity); 0.2 – 0.4; and ≥0.5 (highest acuity) [18].

At follow-up (seven weeks), data were collected on visual function using the VF-14.

The primary outcome measure for this analysis is the VF-14, a valid and reliable 14-item instrument used to assess functional impairment related to vision in both eyes [17,19]. Each item has four possible responses. The questionnaire asks about ability to perform activities of daily living including day and night-time driving, reading small print or traffic signs and engaging in recreational activities. The instrument’s scale ranges from 0 (worst, unable to do all activities) to 100 (best, able to all activities without difficulty).

### Statistical analysis

*Analysis for question 1* (What level of visual impairment was seen pre-operatively in patients scheduled for cataract surgery?)

All patients listed for their first cataract surgery were grouped together into an ‘all first eye’ cohort (n = 142; see Figure 1). This includes patients who only had one surgery in the study period (n = 87) and those who had cataracts removed from both eyes within the study window (n = 55). For all variables collected at baseline, descriptive statistics were calculated.

*Analysis for question 2* (What level of improvement in visual function was seen following cataract surgery?)

Given the focus on the person rather than the eye, this analysis includes only patients who received surgery for both their first and second eye within the study period (‘both eyes’, n = 55). Descriptive statistics were calculated for the post-operative VF-14, and for the change in VF-14 scores (week 7 score minus baseline score).

*Analysis for question 3* (Did gains in visual function following cataract surgery reach the MDC and MCID thresholds?)

Estimates were made of the proportion of patients achieving the respective MDC and MCID threshold levels of 10.81 and 15.57. Given that this analysis is concerned with the change in VF-14, the sample used for this analysis includes only patients who received surgery for both their first and second eye within our data collection time frame (‘both eyes’, n = 55).

At follow-up, not all patients in the ‘both eyes’ cohort had ‘corrected’ vision – some required new reading and/or distance glasses but had not acquired them at follow-up. A sub-group of the ‘both eyes’ cohort was established comprising only those with corrected vision (n = 23). The MDC and MCID thresholds were applied to this sub-group.

## Results

### Sample

Table 1 details the characteristics of participants for both analysis cohorts (‘all first eyes’ and ‘both eyes’). Patients ranged in age from 45 to 94 years, with a mean age of 73, and the majority were female.

**Table 1:** Baseline characteristics of participants scheduled for cataract surgery

	**‘All First Eye’ (n = 142)**	**‘Both Eyes’ (n = 55)**
		**First Eye/Second Eye**
**Baseline Characteristics**	**Mean (SD) or Number (%)**	**Mean (SD) or Number (%)**
Age	72.8 (8.1)	73.0 (7.2)
Female	86 (60.6)	36 (65.5)
**Visual acuity**		
≥0.5 (20/40)	49 (34.5)	28 (50.9)/41 (74.5)
0.2-0.4 (20/100-20/50)	80 (56.3)	24 (43.6)/13 (23.6)
≥0.1 (20/200)	13 (9.2)	3 (5.5)/1 (1.8)
**Glare**		
None	12 (8.6)	3 (5.5)/3 (5.5)
Mild	5 (3.6)	3 (5.5)/2 (3.6)
Moderate	105 (75.5)	36 (65.5)/45 (81.8)
Severe	17 (12.2)	13 (23.6)/5 (9.1)
**Age Related Macular Degeneration**		
None	119 (84.4)	42 (76.4)
Mild	20 (14.2)	11 (20.0)
Moderate	2 (1.4)	2 (3.6)
Severe	0 (0)	0 (0)
**Ocular Comorbidities**		
None	118 (83.7)	42 (77.8)/43 (79.6)
Mild	13 (9.2)	7 (13.0)/7 (13.0)
Moderate	9 (6.4)	4 (7.4)/2 (3.7)
Severe	1 (0.7)	1 (1.9)/2 (3.7)
**Extent of Impairment in Visual Function**		
None	1 (0.7)	1 (1.8)/0 (0)
Mild	16 (11.3)	5 (9.1)/3 (5.5)
Moderate	123 (86.6)	47 (85.5)/52 (94.5)
Severe	2 (1.4)	2 (3.6)/0 (0)
**Ability to Function Independently**		
Not threatened/no difficulties	15 (10.6)	4 (7.3)/3 (5.5)
Not threatened but more difficult	68 (47.9)	44 (80.0)/47 (85.5)
Threatened but not immediately	51 (35.9)	7 (12.7)/5 (9.1)
Immediately threatened or unable	8 (5.6)	0 (0)/0 (0)
**Indications for first/second eye**		
Significant cataract	137 (100)	55 (100)/52 (98.1)
Driving	17 (12.4)	9 (16.4)/10 (18.9)
Needs better view	0 (0)	0 (0)/1 (1.9)
Other medical disease	2 (1.5)	1 (1.8)/1 (1.9)

### Baseline visual impairment levels

**Figure 2:**
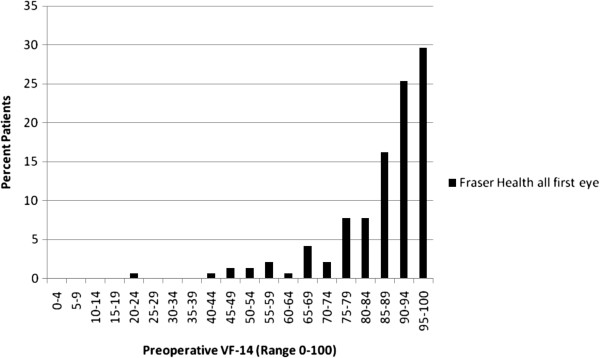
Pre-operative VF-14 for the Fraser Health ‘All First Eye’ cohort (n = 142).

Baseline data on best corrected visual acuity for the ‘all first eye’ group spanned the entire range with 34.5% scoring 0.5 or better, 56.3% scoring between 0.2 and 0.4 and 90.8% scoring 0.2 or better (Table 1). The pre-operative VF-14 scores for the ‘all first eye’ sample are shown in Figure 2. The mean pre-operative VF-14 score was 86.7 (median: 90.9; interquartile range: 14.6). A highly skewed baseline VF-14 distribution is revealed with the vast majority of patients having a score of 80 or higher (on the 0–100 scale). For the ‘both eyes’ group, most patients (74.5%) had better baseline visual acuity in their second eye (Table 1).

**Figure 3:**
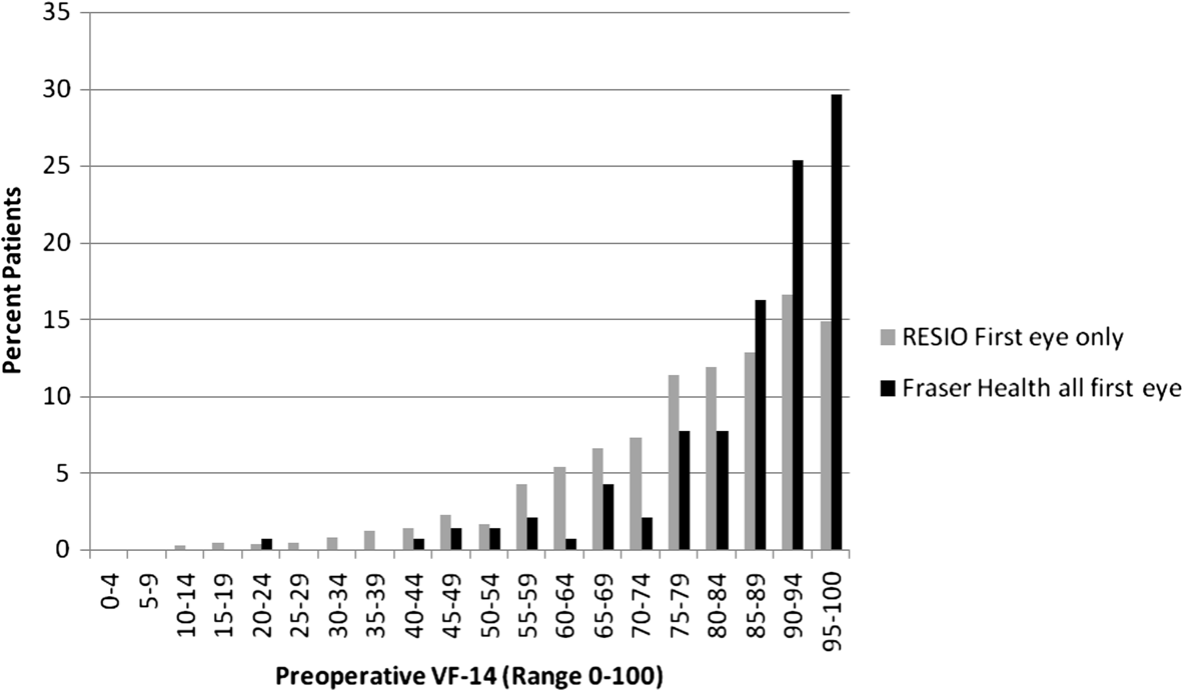
Pre-operative VF-14 for the RESIO cohort (n = 2840) and Fraser Health ‘All First Eye’ cohort (n = 142).

In order to provide a reference point, comparison was made of these new data with those reported by the RESIO investigators a decade earlier (Figure 3). (Note, the RESIO data are also ‘first eye only’.) The distribution of pre-surgery VF-14 scores for the new cohort is further right-skewed compared to RESIO, indicating even higher levels of visual functioning pre-surgery.

### Post-operative visual functioning, change in outcomes and MCID/MDC

**Figure 4:**
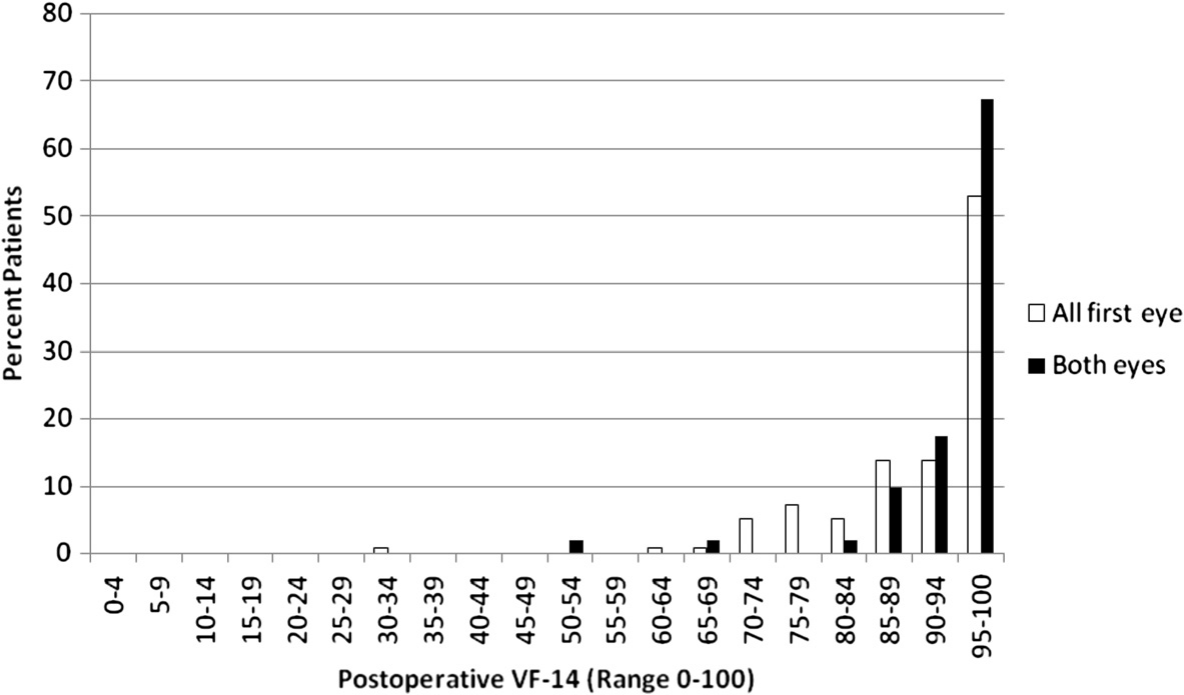
Post-operative VF-14 (‘All First Eye’, n = 142; ‘Both Eyes’, n = 52).

Figure 4 reports the post-operative VF-14 scores. Data are presented for the ‘all first eyes’ and ‘both eyes’ groups.

For the ‘all first eye’ group, the mean post-operative VF-14 score was 92.0 (median: 96.8 interquartile range: 12.5).

For the ‘both eyes’ group, the mean post-operative VF-14 score was 94.8 (median: 97.9, interquartile range: 9.0).

The vast majority of all patients had a post-operative score above 85. The ‘all first eye’ and ‘both eyes’ groups differ at the top end of the scale: in excess of 60% of the ‘both eyes’ cohort report a score of 95 or higher, compared to approximately 50% of the ‘all first eye’ patients. The data highlight that patients in the ‘all first eye’ group do not experience the full magnitude of benefit until their second eye is completed, supporting a focus on ‘both eyes’ in looking at outcomes in this patient group.

**Figure 5:**
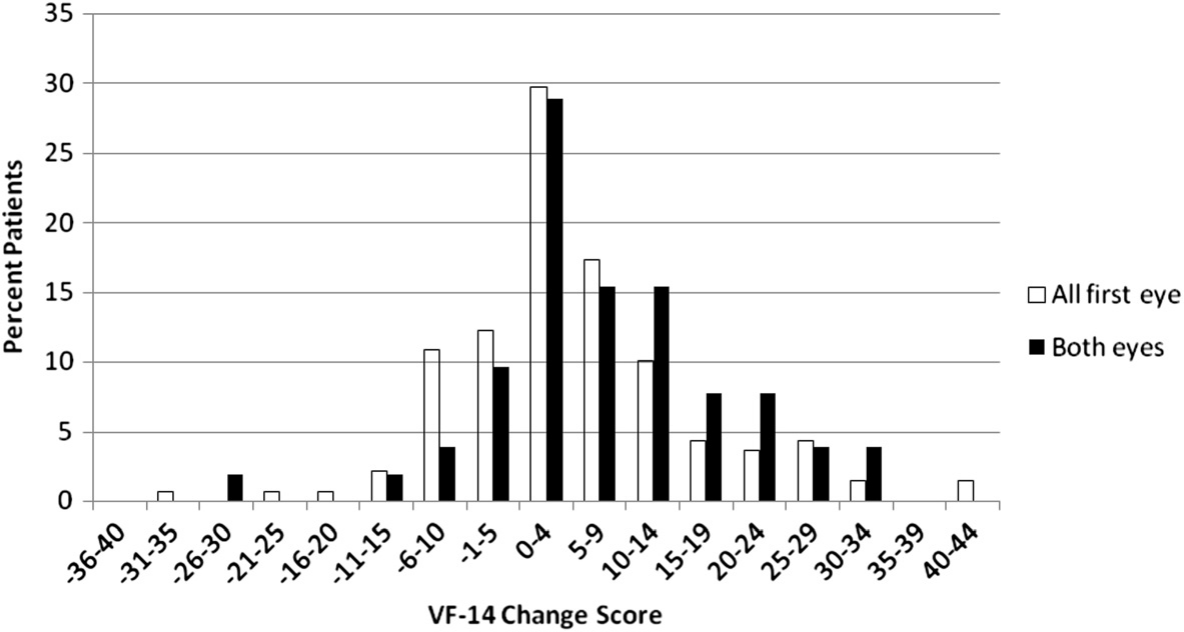
Mean VF-14 Change (‘All First Eye’, n = 142; ‘Both Eyes’, n = 52).

The change in visual functioning (from baseline to seven weeks post-surgery) is reported in Figure 5 and Table 2. For the ‘both eyes’ group, the mean change in VF-14 was 7.5 (standard deviation: 11.3). Despite this average indicating a positive VF-14 change, 17.3% of patients reported a decline in visual functioning post-surgery, and a further 5.8% experienced no change. The remainder, and the majority, of the patient cohort (76.9%) experienced an improvement in visual function.

**Table 2:** Mean VF-14 change scores in ‘Both Eyes’ by clinical indicators of severity

**Baseline Characteristics**	**VF-14**
	**Mean Change (SD)**
Total (n = 52)	7.5 (11.3)
Female (n = 34)	6.4 (12.6)
Male (n = 18)	9.7 (8.3)
Visual Acuity	
≥0.5 (20/40) (n = 27)	4.2 (10.3)
0.2-0.4 (20/100-20/50) (n = 24)	11.5 (12.0)
≤0.1 (20/200) (n = 1)	8.9 (5.7)

When exploring variation in outcomes by baseline visual acuity (Table 2), a trend is evident: larger gains in visual functioning were achieved by patients with poorer baseline visual acuity. This is an unsurprising finding, given the greater scope for improvement amongst those with poorer functioning, but nevertheless important to document.

As indicated earlier, the MCID for the VF-14 is 15.57 and the MDC is 10.81 [14]. For individuals in the ‘both eyes’ cohort, 23% achieved an improvement in visual function that was at or beyond the MCID threshold and 35% saw an improvement beyond the MDC.

For the ‘corrected’ vision sub-group, improvements in visual function beyond the MDC and MCID thresholds were 26% and 39% respectively.

## Discussion

Let us return to our primary study questions.

1 What level of visual impairment was seen in patients scheduled for cataract surgery?

The level of visual functioning pre-operatively is high, with a mean VF-14 score of 86.7 and a highly skewed distribution. The RESIO project, conducted over 10 years ago, highlighted that the pre-operative visual function in Canada was higher than that for other cohorts in the United States, Denmark and Spain [20]. Despite this initial evidence that patients in Canada may be going for surgery earlier than elsewhere, our new data highlight that the distribution may have shifted even further to the right, indicating even higher levels of visual functioning pre-operatively.

2 What level of improvement in visual function was seen following cataract surgery?

The average gain per patient in visual functioning is 7.5 points on the VF-14 scale. However, approximately one in five patients receiving cataract surgery recorded poorer visual functioning after surgery, and a further one in 15 appeared to experience no change. The variation in gains is largely explained by baseline visual acuity: the greatest improvements were seen in those with the poorest acuity levels. This is a finding well supported in the literature [21]. Specifically, one recent study in the UK demonstrated that if the average gain per patient in visual functioning was 5.5 points on the VF-14 scale, 30% of operations would be deemed inappropriate [12]. If average gain per patient in visual functioning was 12.2 points on the VF-14 scale, 49% of operations would be deemed inappropriate. Of note, in this example inappropriateness was defined using threshold for different levels of change in visual function. For example, with a threshold of 5.5 on the VF-14 scale, many patients got past this low threshold and thus few were deemed inappropriate. In contrast, with a threshold of 12.2, fewer patients got to this higher level and thus more were deemed inappropriate. Further, they detailed that the method of determining a clinically important difference strongly influenced the percentage of operations deemed inappropriate [12]. In the quest for the best clinical outcomes, which in turn drives efficiency, it seems important to examine the criteria used for assessing appropriateness of cataract surgery [2-4].

3 Do gains in visual function following cataract surgery reach the MDC and MCID thresholds, and what are the implications of these thresholds for clinical practice in Canada?

The concepts of MDC and MCID are important in making judgments concerning value. We should expect that health gains post intervention achieve at least the MDC; if they do not then we are unable to distinguish between actual change and measurement error. Our hope, of course, is that the MCID threshold for improvement is reached, providing reassurance that patients are routinely experiencing clinically important improvements as a result of surgery. The disappointing finding is that the majority of patients did not meet the VF-14 thresholds of 10.81 and 15.57 for MDC and MCID respectively. The most plausible hypothesis for this finding is that we are not able to observe a MDC or MCID in most patients because of their high pre-operative visual functioning.

Providing refractive correction is necessary to minimize visual impairment in cataract surgery patients [22]. In our sub-group analysis of patients who had experienced vision correction post-surgery, the proportion of patients reaching the MCID and MDC thresholds was only slightly higher than in the overall ‘both eyes’ cohort. Thus, the conclusion that the majority of patients do not reach the thresholds is robust.

In considering the appropriate unit of analysis (i.e., eye or person), we found that the gains in visual function are greater when the analysis focused on those who received cataract extractions in both eyes rather than those who received the first eye only. Often vision is not corrected with eye glasses until after the second eye is completed. Thus, the magnitude of improvement after the first cataract surgery will likely be less and so it seems unfair to assess outcomes for partial procedures. Moving forward, we suggest using ‘both eyes’ (i.e., the person) for analyses of cataract procedures. The VF-14 was designed to be completed by the patient considering their visual functioning with both eyes [18,23]. Therefore, using the person as the unit of analysis aligns with the original intention of the instrument. This recommendation will impact future effectiveness and efficiency estimates for cataract extraction.

### Limitations of the VF-14

The primary limitation of this study may be the sole use of the VF-14. Although the VF-14 was used due to its widespread use in the literature to provide a basis for comparison of our study findings, there is notable skepticism relating to the validity of the VF-14. Given that safety and predictability of cataract surgery have improved over the years, it is possible that the VF-14 is out of date and that other newer instruments are demonstrated as more responsive to cataract surgery [24]. A few reasons why the VF-14 is one of the least responsive instruments may be related to the structure of the questionnaire responses (i.e., not framed to encourage admission of disability) and the instrument noise [25,26]. Although the noise of the VF-14 can be improved through Rasch analysis, we did not include that in this analysis because it would then render it impossible to make meaningful conclusions relating to MDC and MCID values. Thus, our conclusions should be interpreted keeping the limitations of the VF-14 in mind.

This study was conducted as an evaluation and so data were not collected on socio-economic status, other activities of daily living and non-ophthalmologic co-morbidities. The pre-operative visual acuity was collected earlier, at the time that the patient was first scheduled for surgery, than was visual function, which was collected at the time of surgery. As a result, visual acuity may decrease between the time of assessment and immediately prior to surgery. The MDC and MCID analyses should be interpreted with caution, given that the formulation of these thresholds is dependent on the visual functioning of the population used. Finally, the ideal would be to map longitudinal outcome trajectories to assess the potential longer-term benefits associated with early cataract surgery.

## Conclusions

Some patients going forward for cataract surgery have very high levels of visual function. The consequence of this, unsurprisingly, is that clinically important improvements in visual function are not being seen in all patients and so the full value from the intervention is not being gained. This observation speaks to the potential benefits, from a clinical program management perspective, of routine collection and evaluation of thresholds and outcomes data for surgical procedures, such as cataract surgery. Further, future research should continue to focus analyses on the person (i.e., ‘both eyes’) and consider the longer-term trajectory of benefits from cataract surgery.

## Competing interest

The authors declare that they have no competing interests.

## Authors’ contributions

JD, CIHR postdoctoral fellow, and SB, Director of the Centre for Clinical Epidemiology and Evaluation, provided expertise that informed the evaluation design, undertook the data analysis, supported the interpretation of results, and led on drafting of the manuscript. HMN was responsible for evaluation design and data collection. MW, Epidemiologist, Department of Evaluation and Research Services, provided statistical expertise that informed the evaluation design and supported data analysis and interpretation. SC, Director of the Department of Evaluation and Research Services, provided leadership and guidance during the evaluation planning and data collection stage, and supported data interpretation and manuscript preparation. All authors reviewed and revised the manuscript.

## Pre-publication history

The pre-publication history for this paper can be accessed here:

http://www.biomedcentral.com/1471-2318/12/50/prepub
